# Comparative analysis of SARS-CoV-2 detection methods using stool, blood, and nasopharyngeal swab samples

**DOI:** 10.11604/pamj.2023.46.21.39483

**Published:** 2023-09-14

**Authors:** Marceline Adhiambo Oloo, Shehu Shagari Awandu, Benson Onyango, Richard Odongo Magwanga, Alfred Ochieng Oluoch, Shirley Lidechi, Erick Mbata Muok, Stephen Munga, Benson Estambale

**Affiliations:** 1 School of Biological, Physical, Mathematics and Actuarial Sciences, Jaramogi Oginga Odinga University of Science and Technology, P.O Box 210-40601, Bondo, Kenya,; 2 School of Health Sciences, Jaramogi Oginga Odinga University of Science and Technology, P.O Box 210-40601, Bondo, Kenya,; 3 Kenya Medical Research Institute Centre for Global Health Research (CGHR), P.O Box 1578-40100, Kisumu, Kenya

**Keywords:** Severe acute respiratory syndrome corona virus 2, polymerase chain reaction, rapid antigen detection test, enzyme-linked immunosorbent assay, immunoglobulin M, immunoglobulin G

## Abstract

**Introduction:**

as a public health policy, the ongoing global coronavirus disease 2019 vaccination drives require continuous tracking, tracing, and testing of severe acute respiratory syndrome coronavirus 2 (SARS-CoV-2). Diagnostic testing is important in virus detection and understanding its spread for timely intervention. This is especially important for low-income settings where the majority of the population remains untested. This is well supported by the fact that of about 9% of the Kenyan population had been tested for the virus.

**Methods:**

this was a cross-sectional study conducted at the Kisumu and Siaya Referral Hospitals in Kenya. Here we report on the sensitivity and specificity of the rapid antigen detection test (Ag-RDT) of SARS-CoV-2 compared with the quantitative reverse transcriptase polymerase chain reaction (RT-qPCR) using stool and nasopharyngeal swab samples. Further, the mean Immunoglobulin M (IgM) and Immunoglobulin G (IgG) antibody levels among symptomatic and asymptomatic individuals in western Kenya were evaluated.

**Results:**

the sensitivity and specificity of Ag-RDT were 76.3% (95% CI, 59.8-88.6%) and 96.3% (95% CI, 87.3-99.5%) with a negative and positive predictive value of 85% (95% CI, 73.8%-93.0%) and 93% (95% CI, 78.6%-99.2%) respectively. There was substantial agreement of 88% (Kappa value of 0.75, 95% CI, 0.74-0.77) between Ag-RDT and nasopharyngeal swab RT-qPCR, and between stool and nasopharyngeal swab RT-qPCR results (83.7% agreement, Kapa value 0.62, 95% CI 0.45-0.80). The mean IgM and IgG antibody response to SARS-CoV-2 were not different in asymptomatic individuals, 1.11 (95% CI, 0.78-1.44) and 0.88 (95% CI, 0.65-1.11) compared to symptomatic individuals 4.30 (95% CI 3.30-5.31) and 4.16 (95% CI 3.32 -5.00).

**Conclusion:**

the choice of an appropriate SARS-CoV-2 diagnostic, screening, and surveillance test should be guided by the specific study needs and a rational approach for optimal results.

## Introduction

The highly infectious coronavirus disease 2019 (COVID-19), caused by severe acute respiratory syndrome coronavirus 2 (SARS-CoV-2), has disrupted human lives globally [1,2]. Since its first detection [3], it has rapidly spread across the globe exacting a huge toll on human well-being and the economy. Globally SARS-CoV-2 has infected approximately 769 million individuals and has resulted in 6.9 million deaths. Africa has not been spared with several countries reporting ongoing community transmissions with increased infections adding up to a fatality of 9.5 million [4]. Kenya recorded its first case of COVID-19 infection on March 13^th^ 2020 [5]. Since then, it has experienced up to six waves of COVID-19 outbreaks, with diverse variants, *Omicron* being the most dominant variant as of 7^th^ February 2022, which resulted in 342,268 confirmed cases and 5,688 deaths as of December 2022 [6]. However, the fourth wave of infections in June 2021 was characterized by the *Delta* variant, first detected in western Kenya, affecting the counties of Kisumu, Siaya, Homabay, Migori, and Kakamega [7,8]. While containment restrictions and mass testing were introduced, no evaluation of the efficiency of COVID-19 testing regimes adopted by the county´s health departments was conducted.

According to the Centre for Evidence-Based Medicine, nasopharyngeal swab sampling is commonly used for COVID-19 infection testing [9], however, patients find it invasive and uncomfortable [10]. Other forms of human samples used in the detection of SARS-CoV-2 viral presence include stool, urine, and saliva of COVID-19-infected patients [11]. However, the SARS-CoV-2 positivity rates are dependent on a multiplicity of factors including sample type with notable differences between the anatomical collection sites [12]. Thus, a comparison of test outcomes with clinical samples from different anatomical sites can be a practical approach to validate COVID-19 infection, especially in case of variations between clinical symptoms of COVID-19 disease and test results [13].

The antigen-rapid diagnostic tests (Ag-RDTs) recommended by WHO, can aid in rapid SARS-CoV-2 detection and isolation of possible superspreaders prior to confirmatory detection using RT-qPCR [14]. The short-turn-around-time is important for quick health decision-making and allows for screening during pre-operative management for invasive [15]. However, challenges to the ability of Ag-RDT to distinguish between true positives and true negatives abound [16], especially with low virus loads in a swab. In the Kenyan counties of Kisumu and Siaya, tests for COVID-19 are routinely performed with the Ag-RDT from nasopharyngeal swab samples with RT-qPCR as a confirmatory test for positive samples [17]. Variation in the performance of Ag-RDT and RT-PCR is greatly observed between the manufacturers and evaluating their analytical limit of detection. In terms of sensitivity, Ag-RTD is less sensitive compared to RT-PCR, though clinical evaluation of emerging data illustrates that Ag-RDT is accurate at detecting a huge majority of infected individuals with high viral load/cycle threshold on RT-PCR ≤25.0 or >106 genomic viruses [18]. However, test on stool samples for surveillance or detection of SARS-CoV-2 remains largely unexplored in these settings.

The nucleic acid amplification test-based RT-qPCR assay is the gold standard test for laboratory diagnosis of SARS-CoV-2 infection. It is highly reliable with minimal false positive outcomes. However, it´s a complex procedure for poor-resourced labs, relatively expensive, and requires long hours of operation by skilled technicians, limiting its use at a scale [19]. This may hamper efforts to reduce the spread of COVID-19 and lead to the underestimation of prevalence rates [20].

Serological tests are useful in establishing the true spread of COVID-19 and to correlate antibody responses with clinical outcomes [21]. Serology complements the RT-qPCR testing in the later stages of infection and aids in the evaluation of patients´ adaptive immunity status [22]. Thus, previously infected individuals can be identified, even if they were never tested [21], giving a clearer understanding of asymptomatic infections. The serological tests can therefore reveal community transmission of SARS-CoV-2 by identifying individuals who have been exposed and already mounted an immune response against SARS-CoV-2 [23].

Despite the benefits of different SARS-CoV-2 testing regimes, discrepancies still exist in the sensitivity and specificity of these tests using nasopharyngeal, stool, and blood samples [24]. This study determines whether there is a significant difference in the sensitivity and specificity between Ag-RDT and RT-PCR, significant difference between ribonucleic acid (RNA) profiles from the stool and nasopharyngeal swab and the true spread of COVID-19 disease within the two counties. Here we report on the sensitivity, specificity, negative predictive value (NPV), and positive predictive value (PPV) of these tests during COVID-19 surveillance in Siaya and Kisumu counties of western Kenya.

## Methods

**Figure 1:**
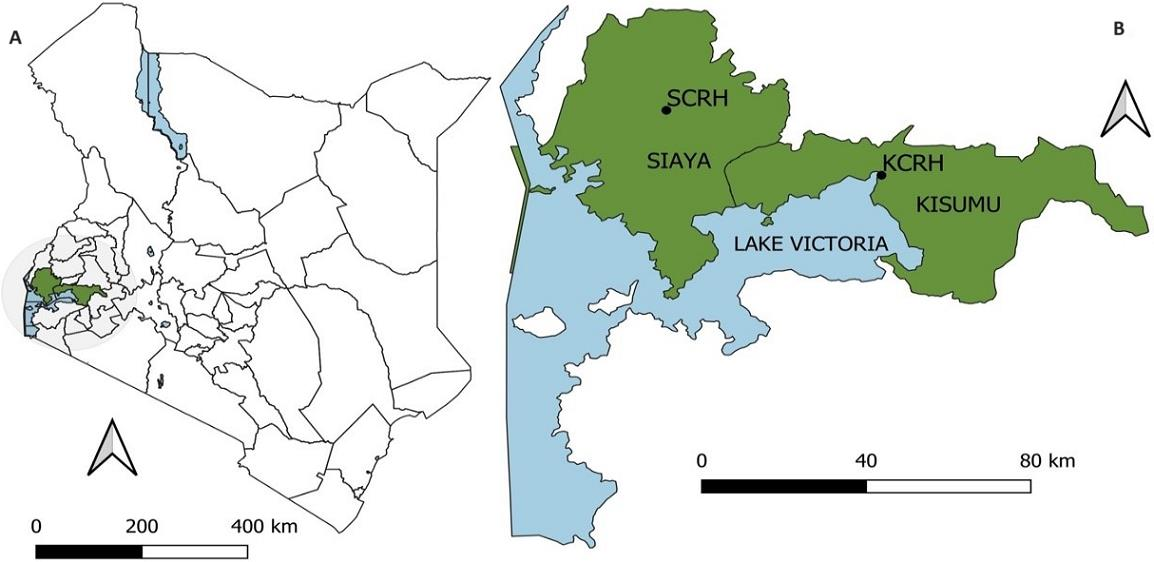
map of the study sites in Kisumu and Siaya counties in western Kenya showing the sampling locations of Kisumu County Referral Hospital (KCRH) and the Siaya County Referral Hospital (SCRH)

**Study and sample design:** a cross-sectional study design was used to obtain samples from Kisumu and Siaya County Referral Hospitals (Figure 1). A non-probabilistic sampling design which is purposive sampling design was used to recruit patients presenting to Kisumu and Siaya Counties referral hospitals for routine COVID-19 tests. All walk-in patients who had presented themselves for COVID-19 screening were eligible for enrollment regardless of COVID-19 symptoms. An informed consent was (explaining the study procedures) provided for the participants to sign upon their agreeing to participate in the study. Personal history was documented on a predesigned form. Demographic data including age, gender, county of residence, patient status, vaccination status and test type whether initial or follow-up/repeat was recorded.

**Study settings and population:** Kenya is located in East Africa, bordering South Sudan to the northwest, Uganda to the West, Somalia to the East, Tanzania to the South and Ethiopia to the North. Kenya is divided into eight regions including Nairobi, the capital city of Kenya, Eastern, Central, Coast, Rift Valley, North Eastern, Western, and Nyanza. Kisumu and Siaya counties are located in the Nyanza province of Kenya. Kisumu is known to be the 3^rd^ largest town in Kenya. These counties are neighboring each other and are located on the shores of Lake Victoria. Kisumu and Siaya have a total population of 2,148,757 (according to the 2019 National Census) accounting for approximately 4 percent of the total population in Kenya. The population consists of both urban and rural dwellers. These counties have two main county hospitals, one in each county, Kisumu County referral hospital is located at a latitude of -0.10054, and at a longitude of 34.75555. Siaya County Referral Hospital on the other hand is located at a latitude of 0.06375, and at a longitude of 34.28707. Fieldwork preparation for this study began on 2^nd^ of November 2021, while recruitment of participants, sample collection and data collection commenced on 25^th^ of November to 22^nd^ of February 2022. Samples were collected at only one time point. Laboratory analysis of the sample were done within the month of March, April and May of 2022. All patients who had presented themselves for COVID-19 screening were eligible for enrollment regardless of COVID-19 symptoms. Upon accepting to participate in the study, all study recruits were taken through and signed informed consent.

**Variables:** independent variables included Ag-RDT (nasopharyngeal swab), RT-PCR (stool, NPs) and ELISA (blood). Controlled variables on the other hand included age, gender, patient status and vaccination status. Positive/negative COVID-19 cases, cycle threshold values, IgM and IgG ratios were the outcome variables.

### Data resource and measurement

**Data collection tool:** a structured case investigation form for the 2019 novel coronavirus (2019 nCoV) adapted from the Ministry of Health, Division of Disease Surveillance and Response of Kenya.

**Data collection:** the patients´ personal history was documented on a predesigned form and included age, gender, county of residence, patient status, vaccination status, and test type whether initial or follow-up/repeat. Data entry was done in an Excel sheet and then exported to STATA software for further analysis.

**Sample size:** sample size was calculated in an online Rao soft platform [25], using a margin of error of 10.19% and with a 95% confidence interval with a 50% response distribution, giving at least 92 samples.

**Sample collection and storage:** sample collection, handling, and storage were done according to protocols described by the Kenya Ministry of Health [25]. Human blood, stool, and nasopharyngeal samples were taken by well-trained health practitioners with the required personal protective equipment (PPEs) to minimize COVID-19 transmission. The nasopharyngeal swabs, blood and stool specimens were then transported to the Kenya Medical Research Institute Centre for Global Health Research Biosafety Laboratory level 3 laboratory for analyses.

### Laboratory assays

**Rapid antigen detection test (Ag-RDT):** the Panbio™ COVID-19 Ag rapid test (Abbott point of care test kits, Germany) were used according to manufacturer instructions. Briefly, 10 drops of buffer were added into a test tube, and a swab tip containing the specimen was then immersed into the buffer then mixed by swirling. At the bottom of the extraction tube, the dropping nozzle cap was opened and 5 drops of the extracted specimen were dispensed into the specimen well. Results were read after 15 minutes. The presence of only the control line (C) and no test line indicated negative results whereas, positive results were determined by the presence of the test line (T) and the control line (C) within the result window. The absence of a control line rendered test results as indeterminate.

**Ribonucleic acid (RNA) extraction and quantitative polymerase chain reaction (qPCR) reactions:** extraction of RNA from nasopharyngeal swabs and stool samples was performed using QIAamp Viral RNA Kit (Qiagen) in accordance with manufacturer instructions. The extracted nucleic acid samples were tested for SARS-CoV-2 presence by RT-PCR using the 7500 fast real-time PCR system (applied biosystems), in accordance with manufacturer instructions. Quantitative Real-time PCR was performed using a DAaN gene SARS-CoV-2 PCR Kit (DaAn Gene Co, Ltd., of Sun Yat-sen University, China). The detection kit for COVID-19 RNA (PCR fluorescence probing) technology was used. Results were analyzed by 7500 fast real-time PCR software version 2.3 to identify SARS-CoV-2 positive targets by evaluating PCR curves for sigmoidal amplification. A sample was considered positive for the targeted pathogen when it had a cycle threshold (CT) value within 40 cycles, negative extraction blank, positive amplification of *N* and *open reading frames (ORF) 1ab*, positive amplification for positive control wells, and fluorescence amplification curves for the internal control well. Samples with no amplifications were retested.

**Enzyme linked immunosorbent assay (ELISA) test:** the IgM and IgG antibodies against SARS-CoV-2 in serum specimens were detected using a quantitative indirect SARS-CoV-2 Detect™ IgG ELISA kit and SARS-CoV-2 Detect™ IgM ELISA kit (InBios International, Seattle, USA), in accordance with manufacturer instructions. The recombinant antigen contained spike protein of SARS-CoV-2. The plates were read on a BIOTEK ELX800 absorbance microplate reader at 450 nm absorbance. The raw optical densities (ODs) were recorded and ratios were computed in relation to the average ODs of the cut-off controls. Samples with IgG or IgM ratio greater than or equal to 1.1 were considered positive and IgG or IgM ratio less than or equal to 0.9 were considered negative. Samples that were neither positive nor negative were classified as indeterminate results.

**Data analysis:** descriptive statistics were used to analyze the data obtained from the participants. Sensitivity, specificity, positive and negative predictive values, Kappa coefficient analysis, and the receiver operating characteristic (ROC) curve were determined. T-test (student t-test) was used to compare the Ct values for the *N* gene and *ORF 1ab* gene between the nasopharyngeal and stool samples and to compare the presence of IgM and IgG between asymptomatic and symptomatic patients. Statistical significance was determined at a P-value of 0.05 or 5%, and confidence intervals were calculated at 95% levels. All analyses were conducted using STATA version 16.

**Ethical considerations:** this study was performed in line with the principles of the Declarations of Helsinki. Approval was granted by Jaramogi Oginga Odinga University of Science and Technology ERC/21/5/21-4. The research license was granted by the Kenya National Commission for Science and Technology NACOSTI/P/22/17543. Administrative approval was provided by the county governments of Kisumu and Siaya.

**Funding:** this work was supported by the National Research Foundation-South Africa under the COVID-19 Africa Rapid Grant Fund (Nr: COV19200616532700). Shehu Shagari Awandu has received support from Africa Research Excellence Fund Research Development Fellowship 2022, (AREF- 312-AWAN-F-C0907). The funders had no role in study design, data collection and analysis, decision to publish, or preparation of the manuscript.

## Results

**Figure 2:**
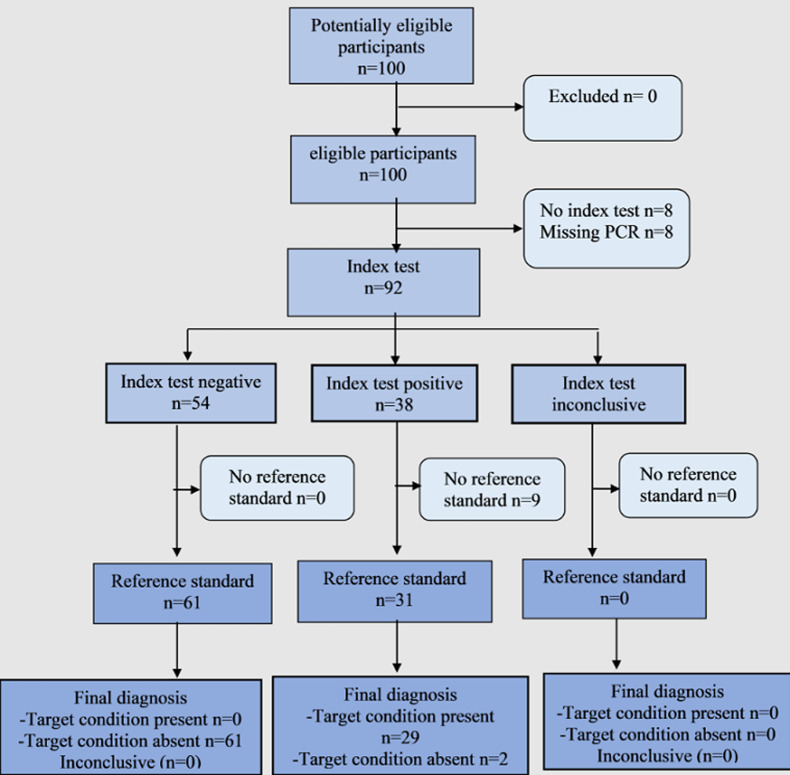
prototypical STARD diagram to report flow of participants through the study

**Participants:** a total of 100 participants were recruited from Siaya and Kisumu County referral hospitals, nasopharyngeal swabs, blood, and stool samples were then collected from each individual. Of the 100 recruited participants, 92 of them had all the data from each variable, while 8 remaining participants who had missing data on PCR were discarded (Figure 2).

**Characteristics of study participants and prevalence of COVID-19 from the different samples analyzed:** this study recruited a total of 92 COVID-19 patients each providing nasopharyngeal swabs, stool, and blood samples. The median age of the participants was 28 (interquartile range 20-39.5) years, with 39% (36/92) males and 61% (56/92) females. We then compared the diagnostic value of (Ag-RDT, RT-qPCR [stool and nasopharyngeal samples], and ELISA for the detection of SARS-CoV-2. Samples were dichotomized into symptomatic and asymptomatic groups. From our observations, Ag-RDT (prevalence 41.3%, 95% confidence interval 31.2- 51.3) was not significantly different (p=0.2864) from nasopharyngeal swab RT-qPCR (prevalence 33.7%, 95% confidence interval 24.2-44.3). The stool sample RT-qPCR (30.43%, 95% confidence interval 21.3-40.9) was similarly not significantly different from nasopharyngeal swab RT-qPCR (p=0.6356). A comparison of the detection of Ag-RDT and nasopharyngeal swab RT-qPCR revealed that out of the 31 qPCR-positive patients, the Ag-RDT correctly classified 29 subjects indicating the individuals having the disease (Table 1). Both the Antigen and the nasopharyngeal qPCR reported a total of 52 cases as negatives and were considered true negatives.

**Table 1:** a comparison of Ag-RDT and nasopharyngeal RT-qPCR outcomes for COVID-19 patients in Kisumu and Siaya Counties, western Kenya

Nasopharyngeal RT-qPCR			
Ag-RDT	Negative	Positive	Total
Negative	52	2	54
Positive	9	29	38
Total	61	31	92

True positive: 29, true negative: 52, false positive: 9. False negative: 2, with RT-PCR as the reference standard diagnostic test for SARS-CoV-2; Ag-RDT: rapid antigen detection test; RT-qPCR: quantitative reverse transcriptase polymerase chain reaction

**Diagnostic performance of Ag-RDT and stool RT-qPCR using the nasopharyngeal RT-qPCR as the reference:** Table 2 presents the diagnostic performance of Ag-RDT and stool RT-qPCR using the nasopharyngeal swab RT-qPCR as the reference standard. The sensitivity and negative predictive value of Ag-RDT was greater than that of stool RT-qPCR. In contrast, the specificity and positive predictive value of the stool RT-qPCR were higher than Ag-RDT. The degree of agreement between Ag-RDT and stool RT-qPCR, as measured by Cohen´s kappa (κ), was substantial (0.75, 95% CI, 0.74-0.77 vs. 0.62 95% CI 0.45-0.80). Similarly, the false positivity rates of Ag-RDT and stool RT-qPCR were both 9.8%, while the false negativity rates of Ag-RDT and stool RT-qPCR were 2.2% and 6.5% respectively.

**Table 2:** diagnostic performance of Ag-RDT and stool RT-qPCR, with respect to nasopharyngeal RT-qPCR as reference

Test variables	Ag-RDT	Stool RT-qPCR
True positive (RT-qPCR)	29(31.5%)	22(23.9%)
False positive (RT-qPCR negative)	9 (9.8%)	9(9.8%)
True negatives (RT-qPCR)	52(56.5%)	55(59.8%)
False negatives (RT-qPCR positive)	2(2.2%)	6(6.5%)
Sensitivity	93.5% (95% CI 78.6-99.2)	71% (95% CI 52-85.8)
Specificity	85.2% (95% CI 73.8-93)	90.2% (95% CI 79.8-96.3)
Positive predictive value	76.3% (95%CI 59.8-88.6)	78.6% (95% CI 59-91.7)
Negative predictive value	96.3% (95% CI 87.3-99.5)	85.9% (95% CI 75-93.4)
Area under receiver operating characteristic curve	0.868(95% CI: 0.79-0.94)	0.857(95%CI:0.72-0.89)
Cohens Kappa (κ)	0.75 (95% CI 0.707-0.868)	0.62 (95% CI 0.45-0.80)
P-value	p<0.001	p<0.001

Ag-RDT: rapid antigen detection test; RT-qPCR: quantitative reverse transcriptase polymerase chain reaction

**Figure 3:**
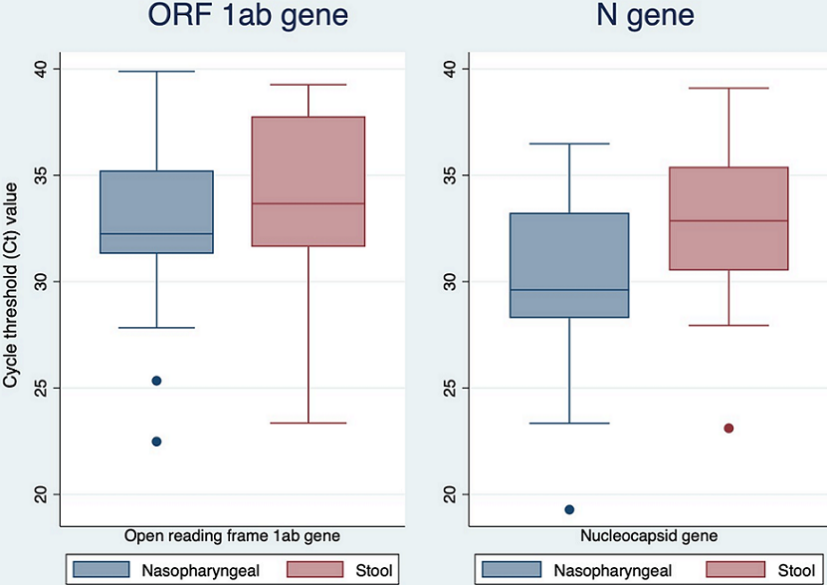
comparison between cycle thresholds for the targeted genes, the *open reading frame 1ab (ORF1ab)* and the *nucleocapsid (N)* in nasopharyngeal swab and stool samples

**SARS-CoV-2 RNA from targeted genes between nasopharyngeal and stool samples:** we compared the *open reading frame 1ab (ORF 1b)* and the *nucleocapsid (N)* genes in the specimen to detect SARS-CoV-2 presence (Figure 3). The mean Ct value for *open reading frame 1ab* did not differ significantly between nasopharyngeal swabs and stool samples (32.46 vs 33.71, p=0.2806). In contrast, the mean cycle threshold value for the *nucleocapsid* gene in the nasopharyngeal swab was significantly lower than that detected in the stool sample (29.54 vs 32.61, p=0.0074).

**Figure 4:**
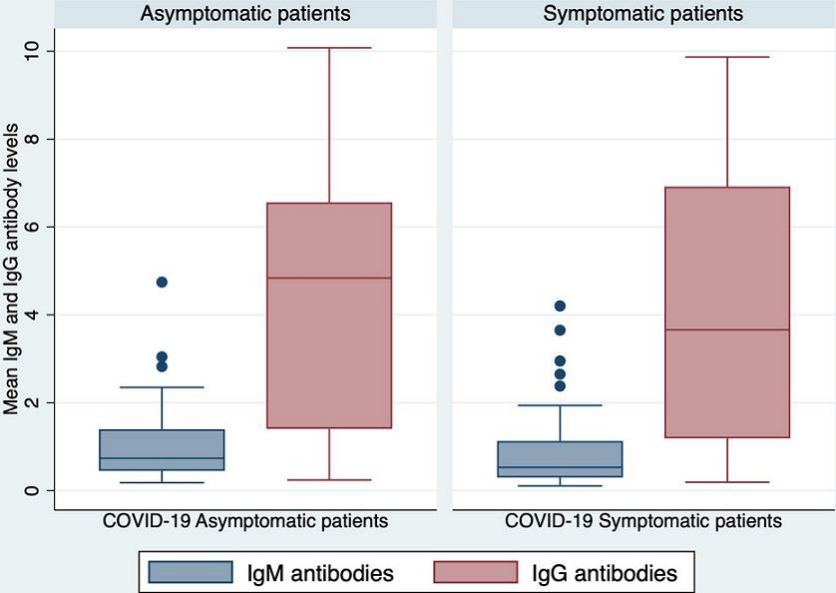
mean IgM and IgG antibody levels in COVID-19 asymptomatic and symptomatic patients

**Mean antibody levels between symptomatic and asymptomatic COVID-19 patients:** we compared the IgM and IgG antibody profiles in the blood serum samples of the participants. The mean anti-SARS-CoV-2 IgM and IgG antibody levels did not differ significantly (1.10 vs 0.88, p=0.2486) and (4.30 vs 4.16, p=0.8315) in asymptomatic and symptomatic patients respectively (Figure 4).

## Discussion

The changing community infection dynamics of COVID-19 pandemic requires reliable diagnostics tests to quickly and accurate identify SARS-CoV-2 infected patients for surveillance and timely containment. The gold standard nasopharyngeal swab RT-qPCR diagnostic test is not without potential preanalytical vulnerabilities and analytical problems [26]. Consequently, alternative diagnostic tests offering advantages in terms of specificity, sensitivity, quick turnaround time and cost effectiveness for mass screenings and population wide surveillance activities are required. In the counties of Kisumu and Siaya of western Kenya, we compared the sensitivity, specificity, PPV and NPV between Ag-RDT and stool RT-qPCR using nasopharyngeal swab RT-qPCR as the gold standard. In these settings, Ag-RDT sensitivity was 93.5% and specificity 85.2% at a cycle threshold value <40. Our findings are similar to observations made in an earlier study in four health facilities in Kisumu County, Kenya [27]. Our results, however, differ with those from the manufacturers which states 99.8% sensitivity and 98.6-100% specificity. Field performance of RDTs kits is variable and can be influenced by several factors including viral load, specimen type, specimen integrity, onset of symptoms, end user competence and other study specific factors [15,28,29]. This may explain the reported 2 false negatives and the 9 false positives, which is a common occurrence [30]. There was a substantial agreement between the Ag-RDT and RT-qPCR indicating consistency in sample collection. Although the Ag-RDTs have lower analytical sensitivity, as point of care tests they can increase access to SARS-CoV-2 screening, confirm early infections, offer quick guidance in health care decisions and can be useful tools to monitor groups at risk of infection [31].

Due to the invasive nature of the nasopharyngeal swab and the associated discomfort during sampling, we investigated whether stool samples could alternatively be used for SARS-CoV-2 detection. Our results indicated that the stool RT-qPCR had a lower sensitivity of 71% and specificity of 90.2% compared to the Nasopharyngeal RT-qPCR. Additionally, the viral load in the stool samples were significantly lower (Ct value 32.61) than in nasopharyngeal swabs (Ct value 29.54). These findings are in line with existing reports of feces containing lower viral RNA loads compared to nasopharyngeal samples dependent on the viral shedding dynamics [32,33]. The variability can be explained by the different virus incubation periods for respiratory and enteric infections, a differing rate of viral replication in each organ/system together with contrasting rates of viral shedding [34]. The average Ct value for *N* gene was significantly lower in nasopharyngeal swabs consistent with its persistent positivity than *ORF 1ab* gene in COVID-19 patients [35,36].

The mean anti-SARS-CoV-2 IgM and IgG antibody levels did not differ significantly between asymptomatic and symptomatic patients. The presence of these antibodies is an indication that majority of the patients may have had COVID-19 infection in the recent past, developing antibodies amidst an ongoing community wide transmission [37]. Studies on SARS-CoV-2 are critical in determining the herd immunity concept. However, when interpreting seroprevalence findings, it´s important to consider the epidemiological moment when each study was carried out. For example, while earlier studies conducted during the pandemic in Kenya recorded lower prevalence of 4.4% [38], more recent studies have found higher seroprevalence of 50.2% [39]. The seroprevalence of IgM and IgG antibodies may also be impacted by the relaxed prevention and control measures including vaccination roll outs. This study was limited by the one-time sampling of the stool samples thus we could not conduct detailed viral shedding studies and the small sample size occasioned by the prevailing COVID-19 situation during the study period.

## Conclusion

High specificity, predictive values and quick results of Ag-RDT implies faster screening of infected individuals to inform better health care decision making. Whilst the viral load in stool was general low, a longitudinal investigation of the viral shedding patterns in patients can elucidate the viral dynamics in stool samples. As a result, the nasopharyngeal RT-qPCR will remain an important tool for SARS-CoV-2 diagnostic and testing that can be augmented by Ag-RDT for mass screening of vulnerable populations.

### What is known about this topic

- *Quantitative reverse transcriptase polymerase chain reaction is known as the gold standard diagnostic test for SARS-CoV-2, in addition, there are several cost-effective diagnostic methods that are being used, for example, the Ag-RDT;*
- *Previous studies have highlighted various sampling regimes for the detection of the novel coronavirus, with nasopharyngeal swabs being the reference sample, few reports indicate that SARS-CoV-2 can be detected in stool;*
- *The use of serological testing has been previously used to determine the true prevalence rates of SARS-CoV-2; such techniques are largely unexplored in Kenya*.

### What this study adds

- *The study elaborates on the high specificity and sensitivity of newly adopted Ag-RDT with the standard reference RT-PCR used in the diagnosis of SARS-CoV-2;*
- *The study contributes new knowledge on the cycle threshold/viral load between stool and a nasopharyngeal swab suggesting that low viral load in stool samples could still transmit SARS-CoV-2;*
- *The study adds knowledge on mean IgG and IgM antibody responses in asymptomatic and symptomatic COVID-19 patients*.
